# Farming thermoelectric paper†
†Electronic supplementary information (ESI) available. See DOI: 10.1039/c8ee03112f


**DOI:** 10.1039/c8ee03112f

**Published:** 2019-01-22

**Authors:** Deyaa Abol-Fotouh, Bernhard Dörling, Osnat Zapata-Arteaga, Xabier Rodríguez-Martínez, Andrés Gómez, J. Sebastian Reparaz, Anna Laromaine, Anna Roig, Mariano Campoy-Quiles

**Affiliations:** a Institute of Materials Science of Barcelona (ICMAB-CSIC) , Campus of the UAB , Bellaterra , 08193 , Spain . Email: alaromaine@icmab.es ; Email: roig@icmab.es ; Email: mcampoy@icmab.es; b City of Scientific Research and Technological Applications (SRTA-City) , New Borg Al-Arab , 21934 , Egypt

## Abstract

Waste heat to electricity conversion using thermoelectric generators is emerging as a key technology in the forthcoming energy scenario. Carbon-based composites could unleash the as yet untapped potential of thermoelectricity by combining the low cost, easy processability, and low thermal conductivity of biopolymers with the mechanical strength and good electrical properties of carbon nanotubes (CNTs). Here we use bacteria in environmentally friendly aqueous media to grow large area bacterial nanocellulose (BC) films with an embedded highly dispersed CNT network. The thick films (≈10 μm) exhibit tuneable transparency and colour, as well as low thermal and high electrical conductivity. Moreover, they are fully bendable, can conformally wrap around heat sources and are stable above 500 K, which expands the range of potential uses compared to typical conducting polymers and composites. The high porosity of the material facilitates effective n-type doping, enabling the fabrication of a thermoelectric module from farmed thermoelectric paper. Because of vertical phase separation of the CNTs in the BC composite, the grown films at the same time serve as both the active layer and separating layer, insulating each thermoelectric leg from the adjacent ones. Last but not least, the BC can be enzymatically decomposed, completely reclaiming the embedded CNTs.

## Abstract 2

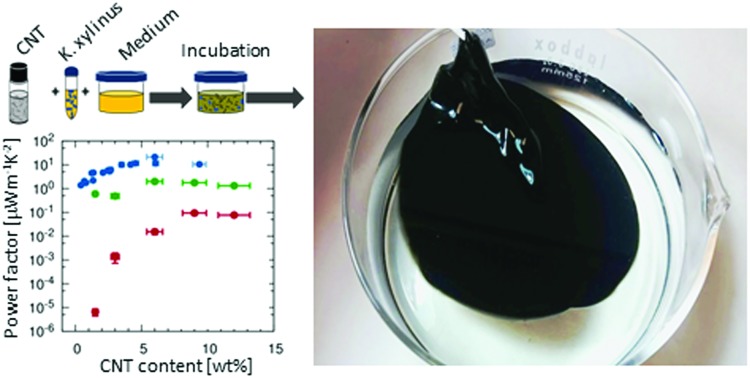
Bacteria are used to grow in an aqueous medium a cellulose-carbon nanotube composite porous film with good thermoelectric properties, flexibility and recyclability.

##

Broader context

The conversion of waste heat into electricity using solid state devices, namely thermoelectrics, has experienced renewed interest in the last decade as novel processing schemes are bringing up performance significantly. Carbon based thermoelectrics, while still exhibiting slightly lower performance compared to their inorganic counterparts, have attracted special attention as they do not contain scarce elements and moreover, can be solution processed at low temperatures increasing sustainability, simplifying fabrication and reducing cost. Here, we go one step further in sustainability and ease of production, by moving from manufacturing to farming thermoelectric films. We let bacteria produce a porous nanocellulose matrix containing a finely dispersed network of carbon nanotubes, in a process more familiar to the preparation of certain Asian desserts. The resulting composite films exhibit comparable thermoelectric properties to buckypapers while saving more than 90% of the expensive carbon nanotubes. In addition, these films are thermally stable beyond 250 °C, amenable to conformal wrapping around arbitrarily shaped heat sources, and importantly, can be enzymatically decomposed to reclaim the embedded carbon nanotubes. This study represents a first step towards a new energy paradigm: do-it-yourself energy materials.

## Introduction

Distributed sources of heat are ubiquitous, and about two thirds of all generated energy is lost in the form of heat. Despite the magnitude of these numbers, this source of energy is not capitalized on because low-grade, distributed heat is unsuited for conventional heat engines. Solid-state thermoelectric (TE) devices, on the other hand, are ideal candidates to exploit this unused heat, as they do not depend on any moving parts and can be scaled nearly arbitrarily, from micro-generation to large scale. While some niche applications exist, the main reason preventing widespread adoption of thermoelectric generators is that they are commonly based on scarce and thus high-priced, often toxic, and brittle inorganic materials. Low cost, flexible and sustainable carbon based materials, which are less problematic concerning toxicity, could potentially become a viable alternative.

Thermoelectric materials are rated using the dimensionless figure of merit, *ZT* = *S*^2^*σT*/*k*, where *S* is the Seebeck coefficient describing the voltage difference that builds up between the two sides of a material held at different temperatures, *σ* is the electrical conductivity, *k* is the thermal conductivity and *T* is the average absolute temperature. Polymers typically exhibit low *k* and hence most scientific efforts are focused on increasing their electronic transport. Novel, highly controlled electronic doping methods have taken conducting polymers from *ZT* of around 10^–5^ to very promising values around 0.2–0.4 in a few years.^1^,^2^ Early success was achieved with stretched, highly doped polyacetylene, which reached a reported power factor of the order of 1000 μW m^–1^ K^–2^.^3^ However, neither the pure, nor the doped material proved to be environmentally stable. Nowadays, the highest performing polymers are PEDOT derivatives.^1^,^2^ The progress on this front has recently stagnated as high doping levels can compromise the mechanical properties of these materials and often result in phase separation of the molecular dopants, inducing brittleness.^4^–^6^ Moreover, doping has been proven most efficient when using sequential methods,^7^–^10^ which due to their diffusion-limited nature, only work well in very thin films (*cf.* highly conductive thick films are required for efficient thermoelectric generators). An alternative to high doping levels consists in adding conductive fillers such as carbon nanotubes (CNTs) to the organic semiconductor matrix;^11^–^16^ an approach that has already successfully yielded *ZT ≈* 0.2.^13^,^17^–^19^ The polymer matrix acts as a binder for the otherwise airborne hazardous CNTs, while providing additional doping paths and a reduced thermal conductivity.^18^,^20^ Conjugated polymers also help in the processing of the CNTs^18^,^20^ due to their favourable interactions, although unfortunately, halogenated solvents are employed in most cases. Besides efficiency, stability is a primary concern, particularly over long periods of thermal stress. This is a major drawback of the existing composites: many semiconducting polymers have relatively low glass transition temperatures (<200 °C), can only work at room temperature and poorly withstand thermal cycling. Moreover, *ZT* (and the overall TE efficiency) will naturally increase if materials could operate at higher *T*.

Different types of polymer matrices have been recently employed in thermoelectric composites, including conjugated polymers^21^ non-conjugated polymers,^22^ water-soluble conjugated polyelectrolytes^19^ and multicomponent polymeric matrices.^6^ While very promising in terms of thermoelectric performance, these systems still lack some desirable traits. An ideal composite should exhibit, simultaneously all or most of the following: capability to be processed from non-toxic solvents using scalable methods to yield large area thick films; consist of an effective dispersion of CNTs that retains good performance even at low CNT loading (thus lowering cost); exhibit some degree of porosity to potentially further reduce composite thermal conductivity and enable access to dopants throughout the thick film;^23^ and significant thermal and mechanical stability under working conditions. Providing a candidate for such a particular matrix is precisely the aim of this work.

Cellulose is a non-toxic, biodegradable and almost inexhaustible biopolymer expected to play a strategic role in replacing petroleum-based polymers. Nanocelluloses are gathering increased interest as they combine the properties of cellulose, such as hydrophilicity, broad chemical-modification capacity and semi-crystalline fibre morphologies, with a large surface area characteristic of nanomaterials.^24^–^26^ Beyond its uses in more conventional sectors such as paper, coatings or food packaging, nanocellulose is being intensively exploited as both an active and passive component in flexible and sustainable electronics,^27^ photonics,^28^ opto-electronics^29^ and energy-related components.^30^–^32^ Nanocelluloses are divided into three classes: cellulose nanofibrils, cellulose nanocrystals and bacterial cellulose.^24^ Bacterial cellulose (BC) is an exopolysaccharide biofilm produced by various microorganisms (*i.e.*, *Komagataeibacter*, *Pseudomonas*). Films are formed by an intertwined network of fibres with fine porosity composed of fibrils with diameters of tens of nanometres, much thinner than plant cellulose. Compared to the latter, BC is characterized by a higher degree of chemical purity, greater tensile strength (derived from a larger degree of polymerization) and a higher degree of crystallinity. A particular benefit of BC is that the generation of composites can be regulated directly *in situ* during the biosynthesis of the polymer, where the hydroxyl-rich nanofibrils promote strong interactions with the surroundings, including solvents, nanoparticles and molecular species. In other words, composites can be farmed and harvested. Very recently, BC composited with PEDOT and PANI showed electrical conductivities in the range of 10 S cm^–1^ for the *in situ* generation of composites,^33^ while 1 × 10^–2^ S cm^–1^ has been reported for composites prepared by filtration of CNTs through an already grown BC membrane.^34^ Given these promising results for the electrical conductivity, as well as other attractive properties of BC composite films, such as the BC low thermal conductivity *k* of around 0.5 W m^–1^ K^–1^,^35^,^36^ there has been surprisingly little research effort to explore the use of these materials in thermoelectric devices, besides the use of paper filters as cheap substrates.^37^

Here we show the *in situ* growth of tens of micrometre thick BC films containing small amounts of well dispersed, percolating single-walled CNTs. The transparency, Seebeck coefficient and thermal and electrical conductivities of the composites (BC/CNT) can be varied by tuning the CNT content. The resulting free-standing conducting papers can be bent up to virtually zero bending radius, as well as conformally wrapped around non-planar objects. Moreover, the BC/CNT composites are thermally stable and can operate even above 200 °C. Indeed, the electrical conductivity increases with increasing temperature, without affecting the Seebeck coefficient. The naturally occurring porosity of bacterial cellulose and the large surface area of its hydroxyl-rich fibrils facilitate the intake of dopants. We successfully n-doped our thick composite films with either polyethylenimine (PEI), NaOH/15-crown-5 ether or tetramethylammonium hydroxide (TMAOH), which reassuringly results in materials exhibiting negative Seebeck coefficients. To demonstrate its potential, we have fabricated a proof of concept thermoelectric paper generator consisting of six p- and n-type pairs of legs.

## Results

**Fig. 1:**
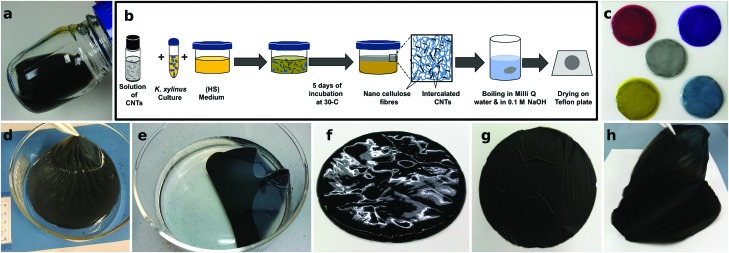
Preparation of BC/CNT composites. (a) CNTs are homogeneously dispersed in the culture medium. (b) Scheme of the preparation steps. (c) Dyed BC/CNT20 composites, each 2 cm in diameter. Large film with a diameter of 11 cm in water, (d) before, and (e) after washing. The insulating top side has a blueish hue. (f) Wet and (g and h) dried film. Final film thickness is about 10 μm for 5 days of incubation.

CNTs are often processed by filtration to form micrometer-thick CNT layers known as buckypaper. These films require large amounts of CNTs and suffer from heavy bundling, which results in suboptimal macroscopic electrical conductivity.^38^ Polymers can aid in dispersing the CNTs to form percolating networks while using less CNTs and reducing the cost of materials. In this respect, bacterial cellulose is unique, as the composite film can be readily grown by culturing the bacteria in media that contain CNTs, *in situ* forming composites that are intertwined at the nanoscale. Fig. 1 details the fabrication protocol used to prepare the BC/CNT composites. A complete description of the process is given in the Experimental section. Briefly, BC films are grown in an aqueous solution of culture medium, in the presence of colloidally stable CNTs. The medium serves the dual function of nourishing the bacteria, as well as dispersing the CNTs. After a culturing time of typically 5 days, the BC/CNT films are harvested, washed and dried, resulting in flexible, square-centimeter-sized and micrometer thick free-standing films. While film thickness can be regulated by the culturing time, the area of the films is dependent on the diameter of the vessel in which the bacteria are grown. Scaling to larger areas is thus as simple as selecting an appropriate vessel, as is demonstrated for the 100 cm^2^-sized films shown in Fig. 1(d–h). Indeed, square meters of bacterial cellulose are regularly farmed by both amateurs and professionals, in a range of industries including, food, design, *etc*. Throughout this work, we will refer to composites prepared from a stock solution as BC/CNT100 (containing ≈15 wt% of CNTs). To study film properties *versus* CNT concentration, this stock solution was further diluted as needed. The BC/CNT composites were labeled as BC/CNT10, 20, 40, 60 and 80 containing 10, 20, 40, 60, and 80% v/v of the stock solution.

These BC/CNT composites can be further processed just as other cellulose-based materials would be. For example, in the same manner as paper, the BC/CNT composites can be easily dyed in a variety of colors without significantly changing their Seebeck coefficient or electrical conductivity (see Fig. 1c). This could enable a whole new range of applications in design or aesthetics, such as thermoelectric wallpaper. To further evaluate the good mechanical properties of the composites, we have recorded the change in relative resistance when bent (ESI† Fig. S1). BC/CNT composites (as well as buckypaper) show no significant change down to the smallest bending radii, which is in good agreement with previous free-standing CNT composites reported in the literature.^39^ This favorable mechanical performance vividly contrasts with the case of highly doped polymers, which are too brittle to be measured when bent, unless they are blended with commodity polymers to improve their mechanical properties.^7^,^40^ The resistance of P3HT/CNT composites is also plotted for comparison. While these conjugated polymer composites are less affected by bending than doped P3HT, their resistance still increases for small bending radii (ESI† Fig. S1d).^18^ The conductivity values of our BC/CNT films are almost unchanged even when maximally bent (a maximum variation of 0.5% was measured). In this respect, while the power factor of our films is still low compared to record inorganic flexible thermoelectrics, such as γ-CuI thin films deposited on PET, the electrical properties of the BC/CNT composites are significantly more robust to mechanical deformation (*cf.* γ-CuI exhibited a 5% change at a 150° angle).^41^ Preliminary data indicate that the tensile strength for both pristine cellulose and BC/CNT is around 73 ± 20 MPa, while the elongation at break is five times higher for the composite (10 ± 3%) compared to the BC (2.3 ± 0.4%).

Furthermore, by humidifying a dry BC/CNT film, cellulose fibers become even more flexible without disrupting the integrity of the whole film. Thus, conformal well-adhered BC/CNT films can be fabricated if never dried or re-wetted samples are allowed to dry in contact with surfaces such as glass. This results in a lower thermal boundary resistance between the heat source/substrate and the TE material. Recently, thermal boundary resistance has, indeed, been found to be as critical as the material *ZT* in defining the capability of a thermoelectric generator to harvest heat.^42^ In a simple set-up, two BC films were fixed on identical surfaces. One was conformally well-adhered to the surface while the other was a dry film fixed with adhesive tape. A heat source was placed on the bottom and the increase in temperature on the surface was monitored using an IR camera (ESI† Fig. S2d and e). Heat transfers faster to the conformal film increasing its temperature higher than the taped sample, which is attributed to empty spaces between the film and substrate. It is worth noting that this protocol to obtain conformal films does not require the use of elevated temperatures, as is the case for the recently reported inorganic conformal inks.^43^

BC is a biodegradable polymer and thus a potential pathway towards reclaiming the more expensive part of the composite (CNTs) could involve enzymatic decomposition. We have exposed a 40% composite film to the enzyme cellulase in a sodium acetate–acetic acid buffer solution and we were able to completely remove the BC, leaving a barely self-supporting, sparse film of recycled CNTs. This suggests an effective recycling method for the more expensive and potentially contaminating CNTs once the generator lifetime has passed. In another experiment, we decomposed several composite samples and reclaimed the CNTs. The dispersion could then be used to form more composites. Here, we simply filtered it to make a thick buckypaper for further characterization (see below).

**Fig. 2:**
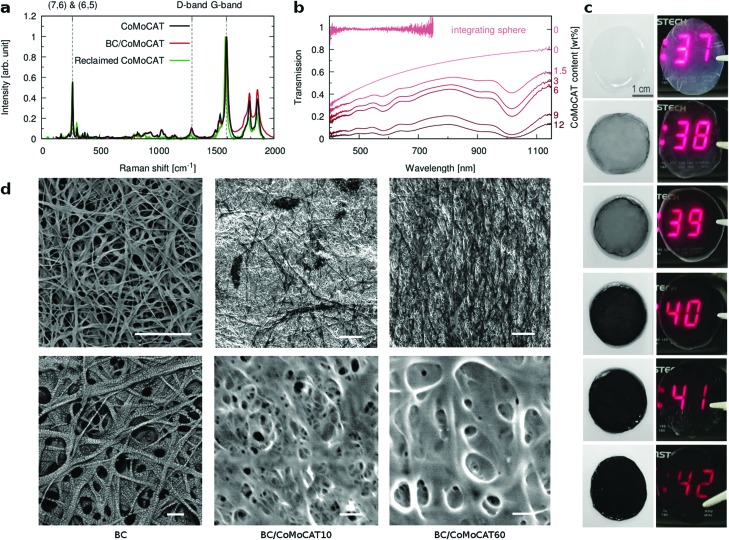
Physical characterization of BC/CoMoCAT composites. (a) Raman spectra of a representative BC/CoMoCAT40 composite as well as of neat and reclaimed CNTs. The G- and D-bands, as well as the radial breathing modes, assigned to specific chiral vectors, are indicated. (b) Transmission of BC and composite films with increasing CNT loading. Neat BC is measured twice, with and without an integrating sphere to highlight the effect of scattering. (c) Photographs of the same samples, ranging in loading from 0 wt% to 12 wt%. (d) SEM micrographs for neat BC and two composites. Scale bars are 1 μm and 100 nm in the top and bottom row respectively.

We next present the structural properties of the composites as studied using microscopy and spectroscopy. The Raman spectra of the BC/CNT composites, of neat CNTs, as well as of CNTs reclaimed after the cellulase treatment are compared in Fig. 2a for CoMoCAT CNTs. All of them exhibit well defined radial breathing modes, which are assigned to CNTs of distinct chiral vectors. Particularly prominent is the 260 cm^–1^ peak, corresponding to a CNT mixture enriched in (6,5)-tubes, as expected for CoMoCAT tubes. We initially chose CNTs rich in semiconducting species, as this was shown to lead to higher thermoelectric performance.^44^ The fabrication protocol does not seem to preferentially select nanotubes of any specific chiral vector. Similarly, the ratio between the G- and D-bands does not change significantly, suggesting that no defects are introduced into the CNTs during the growth or recycling of the bacterial cellulose. Furthermore, Raman maps of whole films confirm that CNTs of different chiral vectors are distributed uniformly throughout the film (not shown).

The transmission spectra of the composites (Fig. 2b) show the characteristic absorption bands between 580 nm and 1010 nm for CoMoCAT CNTs, in agreement with the literature.^16^,^45^ While some transparency is retained in all of these thick films, transmission is clearly reduced with higher CNT loading. Transmission of neat BC, measured with and without an integrating sphere is also shown in Fig. 2b, demonstrating that BC itself is transparent, yet strongly scatters light in the visible region. Complete transmission spectra of all films measured with an integrating sphere are shown in the ESI† Fig. S3. Comparing both measurements, we can estimate that our BC and BC/CNT films scatter between 40% and 60% of the incoming light. Fig. 2c pictures these samples, demonstrating the transparency of the BC films, as well as the fact that they scatter light, resulting in some degree of haze. Light scattering might serve as a light trapping mechanism when using the sun as a source of heat or for bolometric applications. Alternatively, CNT loading could be reduced by using longer CNTs, resulting in more transparent films.^38^,^46^ The origin of the light scattering is due to the porous microstructure of this type of nanocellulose. Fig. 2d shows scanning electron micrographs of BC and of two composite films (BC/CoMoCAT10 and BC/CoMoCAT60). The images reveal a highly porous material with fibers that are about 100 nm in diameter. BC and CNTs form similar fibers or bundles of fibers. Due to their conductive nature, however, CNTs do not electrostatically charge, and appear darker in the lower magnification images signalling the percolating, conducting pathways.

### Electrical characterization

**Fig. 3:**
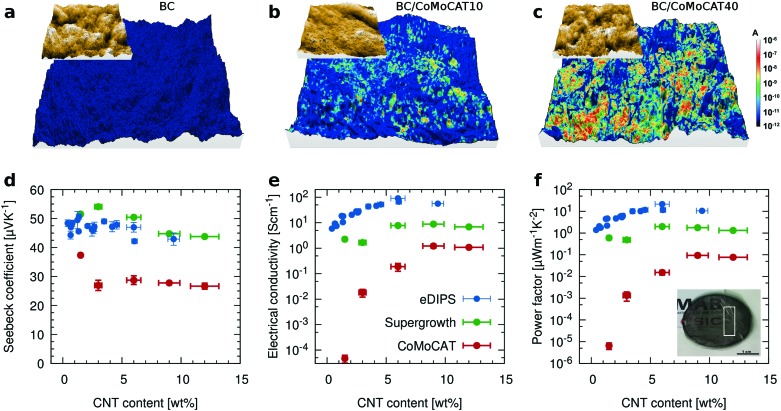
Thermoelectric characterization of the composites. Conductive AFM images of (a) BC film, (b) BC/CoMoCAT10 and (c) BC/CoMoCAT40; macroscopic measurements of the (d) Seebeck coefficient, (e) electrical conductivity and (f) power factor *versus* CNT loading for CoMoCAT (red), Supergrowth (green) and eDIPS (blue) CNTs. The inset shows a representative film, with the white rectangle outlining a homogeneous part used for typical samples.

In order to correlate the morphology with the electrical properties, we have performed conductive AFM measurements using a methodology specifically designed for rough soft matter (see Methods for details). The data are summarized in Fig. 3 and they include the reference BC as well as two composites with different CNT loadings. In the topology images, the fibrillar structure is clearly seen. As expected, the BC film is electrically insulating. By increasing the CNT loading, a greater number of percolating pathways are established, increasing the overall conductive area. To further investigate if the CNTs are indeed well dispersed in the plane of the film, and the ground electrode was not short-circuiting through the film, we measured conductive AFM maps placing the conductive tip at increasing lateral distances from the ground electrode. As documented in the ESI† Fig. S4, no change is observed, confirming a well dispersed network of CNTs percolating over macroscopic distances.

Particularly interesting for thermoelectric applications, is our observation that the films exhibit vertical phase segregation, with the CNTs concentrated on the bottom side of the film. BC is grown in a bacterial culture where the bacteria tend to thrive on the surface for oxygen access. They produce BC films by growing upwards layer by layer. As a result, the top surface of the films is insulating, while the bottom part is highly conductive. Confocal Raman spectra taken at increasing focus depth, confirmed this phase segregation (not shown). SEM images of the top side appeared highly charged, while macroscopic conductivity measurements and conductive AFM further demonstrate that one of the surfaces is conductive and the other is insulating (ESI† Fig. S4c). As shown below, this phase separation proved useful to simplify the fabrication of a prototype generator, utilizing the non-conductive surface as an in-built insulating layer, which separates adjacent p- and n-type legs.

Next, we evaluate the macroscopic thermoelectric performance of these films for 3 different types of CNTs, the CoMoCAT tubes discussed until now, as well as Supergrowth and eDIPS CNTs (Fig. 3d–f). As expected, the electrical conductivity *σ*, increases with increasing CNT loading until it eventually reaches a plateau value. For all three types of tubes, this value is about one order of magnitude lower than for films prepared from neat CNTs. This decrease is attributed to the insulating nature of the BC and the existence of the aforementioned underlying substrate of pure BC, which leads to an overestimation of the “active” thickness. It is interesting to note that the films contain only about 10 wt% of CNTs, which indicates that the BC is effectively helping to disperse the CNTs, as smaller bundles show higher conductivities.^38^,^46^ In other words, if we were to normalize the thermoelectric performance by the amount of CNTs, the composites would outperform the buckypaper. The Seebeck coefficient shows little variation as one would expect from a composite where only one of the two materials exhibits electronic transport. If at all, the Seebeck slightly decreases with increasing CNT loading. The highest measured power factor is of the order of 20 μW m^–1^ K^–2^ for BC/eDIPS composites with a CNT loading of about 6 wt%. eDIPS as well as Supergrowth CNTs to some extent, improve mainly the electrical conductivity due to an improved percolation, compared to the shorter CoMoCAT tubes.

### Thermal characterization

**Fig. 4:**
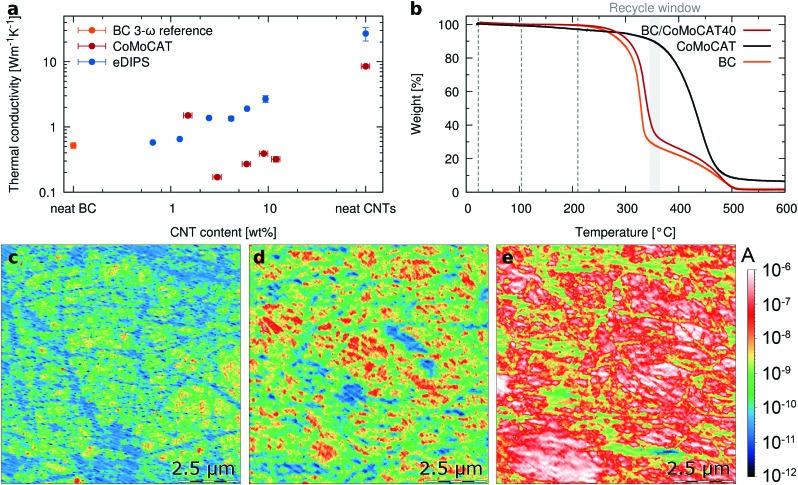
Thermal properties of BC/CNT composites and conductivity at high temperatures. (a) In-plane thermal conductivity measured using Raman thermometry on free-standing films in a vacuum and the 3ω method for BC reference. (b) TGA thermograms for BC, CoMoCAT CNTs and a BC/CoMoCAT40 composite. Conductive AFM maps of BC/CoMoCAT40 measured at temperatures of (c) 24 °C, (d) 104 °C and (e) 210 °C.

We now turn to the thermal properties, and in particular, we are interested in determining if the characteristic low thermal conductivity of the porous bacterial cellulose of about 0.5 W m^–1^ K^–1 ^35,36 is retained in the composites. To avoid misinterpretations related to possible anisotropic behavior,^19^ we have measured the thermal conductivity in-plane by using Raman thermometry.^47^ This method uses a laser to simultaneously excite the resonant Raman signal and heat the sample. After calibration, shifts in the Raman peak positions upon illumination determine the temperature that the sample acquires by absorbing the photon energy. For free-standing films, the temperature rise is then correlated with the in-plane thermal conductivity (see the ESI† for full details). The pure CoMoCAT CNT free-standing films show a thermal conductivity approaching 10 W m^–1^ K^–1^, in agreement with typical literature values for semiconducting CNT mats.^48^,^49^ Reassuringly, compared to buckypaper the thermal conductivity of the BC/CNT composites is decreased by a factor greater than 20 to values similar to those of polymers, *i.e.* around 0.3 W m^–1^ K^–1^ (Fig. 4a). This improvement compensates in excess the loss in electrical conductivity by mixing, making the composite a better material in terms of overall thermoelectric properties even when the amount of active material is less than 10%. The slightly higher *k* for the BC/CoMoCAT10 sample is an outlier, attributed to an inhomogeneous sample. For eDIPS CNT based composites, the trend is the same, a significant decrease from 25 W m^–1^ K^–1^ to about 2 W m^–1^ K^–1^ for the BC/eDIPS system. It should be noted that for neat BC, Fig. 4a reports the out-of-plane thermal conductivity, which was measured using the 3ω method, since BC is transparent and thus cannot be measured using Raman thermometry. Further details are given in the ESI.†

While CNTs are rather thermally stable materials, the matrix polymers commonly used are not necessarily so, which strongly constrains possible thermoelectric applications of CNT composites, due to the narrow operational temperature range of the synthetic polymer matrices. To get an estimate of the maximum potentially usable temperature ranges, we looked at the thermal decomposition temperatures of the composites. Fig. 4b shows the TGA thermograms for BC, CoMoCAT CNTs and a BC/CoMoCAT40 film. The minima of the derivatives of the TGA curves for the three samples are 330 °C, 339 °C, and 443 °C, respectively. This would indicate good thermal stability of the composites for temperatures beyond 250 °C. Interestingly, the temperature window between the complete BC degradation and the onset of CNT degradation offers yet another approach to recycle the CNT in the composites.

To investigate if the thermoelectric properties of the composites are also retained upon heating, we have measured conductive AFM maps at room temperature, 104 °C and 210 °C (Fig. 4c–e). The electrical conductivity of the composite increases with temperature, as expected for a semiconductor. Interestingly, areas of the composites that were almost non-conductive at room temperature become highly conductive with temperature. Histograms of the images as a function of temperature quantitatively demonstrate that most of the material becomes conductive and the average conductivity increases more than one order of magnitude (ESI† Fig. S6). Reassuringly, macroscopic measurements of the resistivity show the same trend (ESI† Fig. S7), while the Seebeck coefficient is retained at higher temperatures. To confirm the stability of the composites, BC/eDIPS samples were annealed at 200 °C for nearly 350 hours. As shown in the ESI† Fig. S8, no significant changes in *S* or *σ* are observed, apart from an initial increase in electrical conductivity. This increase in electrical conductivity above 100 °C is related to desorption of about 5 wt% water, present in the BC/CNT system at 50% relative humidity. This desorption is accompanied by the loss of some of the inherent flexibility of the BC. The figure of merit is plotted in the ESI† Fig. S9. At room temperature, for composites containing 4 wt% eDIPS, we obtain *ZT* > 2 × 10^–3^. Further increase in performance could be achieved by controlling the doping level of the CNTs, as recently reported by MacLeod and coworkers.^50^

### Doping of composites

**Fig. 5:**
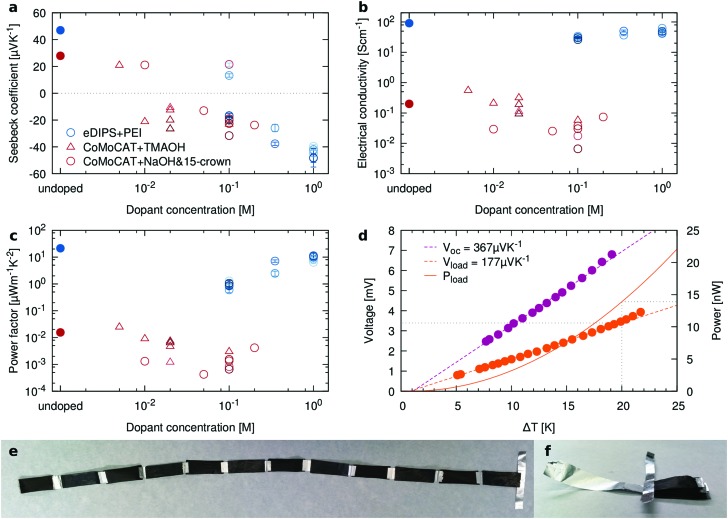
Towards farming of paper-based thermoelectric generators. n-type doping of BC/CNT40 (a) Seebeck coefficient, (b) electrical conductivity and (c) power factor for undoped composite samples (filled circles), and for samples doped with PEI (blue), NaOH/15-crown (red circles) or with TMAOH (red triangles) at increasing concentrations. For a given concentration, samples were doped for increasing times, corresponding to darker colored circles. (d) Voltage and power of a TE module composed of 6 pairs of legs. (e) Extended TE module, as well as (f) folded up into the final zig-zag shape. 1 by 3 cm^2^ sized legs were cut from BC/eDIPS films, and every other leg was n-type doped with PEI before assembly. Legs are connected in series with silver paste and aluminium foil, and no additional insulating layer was used to separate adjacent BC films.

The film porosity can provide a physical pathway for dopants in solution to access the bulk of the material, as compared to solid films in which doping is often diffusion limited.^23^ This is particularly important for technologically relevant thick films. We have explored this potential benefit by n-doping the BC/CNT composites with either polyethyleneimine (PEI), a crown ether complex salt (NaOH & 15-crown-5) or with tetramethylammonium hydroxide (TMAOH)^51^ by immersion, as described in the Experimental section. The results are reported in Fig. 5a–c. While only relatively small changes in *σ* are observed, samples treated with higher dopant concentrations clearly exhibit n-type behavior, with the power factor of doped samples nearly reaching the level of the corresponding p-type control samples.

Fig. 5(e and f) show a simple TE module composed of six pairs of legs which yields a total (open circuit) Seebeck voltage of 367 μV K^–1^, corresponding to approximately 30 μV K^–1^ per leg. When connected across a load that matched its internal resistance of 827 Ω, it generates 177 μV K^–1^, slightly less than the expected amount of half the open circuit voltage. At an applied temperature gradient of 20 K, this corresponds to a generated power of 14.5 nW, as shown in Fig. 5d. Water soluble polyvinyl alcohol was used to paint the exposed aluminum foil, thereby preventing any metal-to-metal short circuits. Besides that, no additional insulators were used to separate neighboring thermoelectric legs of the module. Instead, the insulating side of the BC film provided the necessary insulation, even when the legs were clamped between copper blocks during measurement.

## Conclusions

We present a thermoelectric paper made of finely intermixed bacterial nanocellulose and carbon nanotube fibers. The bacteria-grown films are flexible, porous and thick, and exhibit a thermoelectric performance similar to or even better than that of buckypaper, while containing only about 10 wt% of CNTs. The electrical conductivity of these films does not change upon bending to the smallest bending radius (*cf.* doped conjugated polymers), and they can be conformally wrapped around intricately shaped heat sources, greatly improving heat transfer. The high porosity of the composites keeps the thermal conductivity low (≈1 W m^–1^ K^–1^), while at the same time, it enables easy access of dopants throughout the film thickness for samples with macroscopic dimensions, as we show by n-doping the composites and fabricating a proof of concept TE generator. Importantly, BC/CNT composites are stable from room temperature to, at least, 200 °C. Operation at higher temperatures naturally increases *ZT* and allows for a wider range of viable applications.

BC/CNT composites are environmentally friendly. No scarce elements are employed (*cf.* Bi, Pb, Te, *etc.* contained in inorganic thermoelectrics), the processing solvent is water as opposed to the organic solvents typically used for conducting polymers and the bioproduction of bacterial nanocellulose is not dependent on oil reserves. Moreover, cellulose can be decomposed using temperature or enzymes thus enabling easy CNT recycling. Additionally, the cellulosic matrix acts as a binder to keep the CNTs in place, stopping them from becoming a respiratory hazard (a potential concern for airborne CNTs).

The range of potential applications for organic thermoelectric generators could be broadened considering that these films are stable at medium temperatures, extremely flexible and have tunable appearance, including variable transparency and color. Further thermoelectric improvements are foreseen by continuous feeding of CNTs during BC growth to create thicker films, using CNTs of a single, specific chiral vector and exploring further dopants and doping mechanisms.

## Methods

### Sample preparation

#### CNT dispersions

SG65i CoMoCAT single-walled carbon nanotubes (CNTs) were purchased from Southwest NanoTechnologies and used as-received. Supergrowth nanotubes were kindly provided by Zeon Nano Technology. eDIPS CNTs were bought from Meijo Nano Carbon. Concentrations of 0.45 mg ml^–1^, 0.1 mg ml^–1^ and 0.2 mg ml^–1^ of CNTs were used as the starting solution for CoMoCAT, Supergrowth and eDIPS tubes respectively. The CNTs were dispersed in the Hestrin-Schramm liquid medium (HS) (w/v; 2% d-glucose, 0.5% peptone, 0.5% yeast extract, 0.115% citric acid, 0.68% Na_2_HPO_4_·12H_2_O).^52^ Supergrowth and eDIPS CNTs were tip-sonicated at 200 W for up to 60 min in an ice bath. All CNT dispersions were then autoclaved at 121 °C for 20 min, before being bath-sonicated for 2 h upon cooling down. Afterwards, the dispersion of CoMoCAT tubes was left undisturbed for 3 days at 4 °C allowing larger undispersed aggregates to settle down. For CoMoCAT tubes, only the supernatant containing stable and well dispersed CNTs was used for further processing. By visual inspection we determined that the supernatant contains approximately half of the initial concentration. In the case of the tip-sonicated Supergrowth and eDIPS tubes, no sedimentation was observed and everything was used. The CNT content was calculated from the known weight of CNTs in solution and the measured weight of the final dried films.

#### Inoculum culture

*Komagataeibacter xylinus* was the cellulose-producing bacteria strain purchased from the Spanish type culture collection (Coleccion española de cultivos tipo (CECT)). To make an active culture of *K. xylinus*, we prepared sterile 5 ml aliquots of HS liquid media and inoculated each with a loopful of a bacterial culture previously grown on HS agar media plates. The inoculated aliquots were capped and incubated at 30 °C for 7 days in the dark. At the end of the incubation time; a thick cellulose film is formed on the top of the culture and the liquid culture beneath was used as the inoculum.

#### Composite fabrication

The BC/CNT composites were produced by mixing fresh sterile HS liquid medium with the CNT solution and with 10% v/v liquid of *K. xylinus* culture as inoculum. To produce a series of BC/CNT films with different concentrations of nanotubes, we mixed the dispersed CNTs with the other constituents in varying proportions.

The dispersions were incubated in the dark at 30 °C for 5 days, yielding bacterial cellulose BC/CNT nanocomposite films. To eliminate the culture debris and bacterial cells, the films were collected and immersed in a solution of alcohol and Milli-Q water (1 : 1) for 10 min and then transferred to fresh boiling water for 20 min twice before washing them with 0.1 M NaOH twice. The wet films were collected and rinsed with MilliQ water repetitively until neutral pH indicated the total removal of NaOH. For drying, each film was spread out on a Teflon plate and left to dry at 25–40 °C. The resulting nanocomposites are films in the form of disks, from 25 mm to 110 mm in diameter and about 7–12 μm thick. The thickness of the films was measured with a digital micrometer (Mitutoyo High-Accuracy Digimatic Micrometer). The average sample thickness was determined by measuring at 10 different sites for each film. For n-doping of BC/CNT composites either trimethylammonium hydroxide (TMAOH), (NaOH : 15-crown-5 ether 1 : 1) or polyethyleneimine (PEI), was used. Solutions of 5, 10, 20, and 100 mM of TMAOH were prepared by adding TMAOH in *N*,*N*-dimethylformamide. Never-dried films of BC/CNTs were first immersed in *N*,*N*-dimethylformamide for 6 h, exchanging the solvent every 2 h, then the films were immersed in the TMAOH solutions for 20 min before drying them. 15-Crown-5 solutions of 10, 50, 100, and 200 mM were prepared by dispersing the 15-crown-5 in the aqueous solution with NaOH (1 : 1). Films of BC/CoMoCAT40 were immersed in the solutions for 1 h before drying them. Dry films of BC/eDIPS40 were immersed in 0.1, 0.35 and 1 M PEI in ethanol from 10 s to 1200 s before annealing them at 100 °C for 60 min. BC/CoMoCAT40 films were exposed to the enzyme cellulase in a 0.05 M (pH 5.5) sodium acetate–acetic acid buffer solution for 1 h, 3 h and 6 h to evaluate recyclability.

### Mechanical properties

Pristine bacterial cellulose and bacterial cellulose/CoMoCAT10 composite dry films were cut in 20 × 50 mm pieces, then each specimen was weighed and the superficial mass calculated. Tensile strength and elongation tests were carried out using a 707176 Zwick Z2.5 Dynamometer with a load cell of 2.5 KN. Clamps of pneumatic flat rubber and metal separated 20 mm were used with a 0.05 N preload, a pressure of 6 bar and moving at a constant velocity at 100 mm min^–1^. For each sample, five replicates were measured.

### Electrical characterization

Electrical conductivity (*σ*) was measured using the van der Pauw method^53^ by contacting square 10 × 10 mm^2^ pieces at the corners with silver paste. To measure the Seebeck coefficient *S* of each BC/CNT film, a rectangular piece of approximately 10 × 20 mm^2^ with excellent CNT dispersion homogeneity was cut from each disk (see inset of Fig. 3f). The average Seebeck coefficient between 310 K and 350 K was measured in an ambient atmosphere using a custom built setup. Opposite sides of the sample were placed on a heater and a heat sink respectively, and contacted electrically with silver paste. The temperature gradient between the two contacts was slowly ramped from 0 K to 40 K and back at a speed of about 2 K min^–1^. The temperature at both ends was measured with two k-type thermocouples, while the Seebeck voltage was recorded with a Keithley 2400 SourceMeter. *S* was extracted from a straight line fit of the measured slope.

### Thermal conductivity measurements

One laser Raman thermometry (1LRT) experiments were performed in backscattering geometry using a LabRam HR800 spectrometer equipped with a liquid-nitrogen cooled charge coupled device (CCD) detector. A 488 nm line from an Ar^+^-ion gas laser was used as an excitation source. The temperature increase on the sample was measured as a function of laser power and compared to the experimentally determined absorbed power in order to deduce the in-plane thermal conductivity. The samples were free-standing and held in a vacuum during measurements to avoid convection and photodegradation. Full details are given in the ESI.† Because BC is transparent, the thermal conductivity of the neat BC reference film was measured using the 3ω method, as detailed further in the ESI.†

### Structural and spectroscopic characterization

The microstructure of bacterial cellulose and BC/CNT nanocomposites was investigated using an FEI Magellan TM XHR 400L high resolution scanning electron microscope (SEM) operating at 1 kV using a through lens detector and low-kV high contrast detector. The nanotube containing samples were conductive enough to be visualized as obtained while the BC control specimen was sputtered with 1 nm of gold before investigation.

The BC/CoMoCAT40 composite was selected for thermogravimetric analysis along with the BC film and CoMoCAT powder. The analysis was carried out using a TGA-DSC/DTA analyser (NETZSCH STA 449 F1 Jupiter) at a temperature range from room temperature to 750 °C and heating rate 10 °C min^–1^ in an air atmosphere. The samples of BC/CoMoCAT40, BC, and CoMoCAT weighed 1.75, 1.59, and 1.74 mg respectively.

The current sensing atomic force microscopy (CSAFM) characterization setup was modified in order to avoid friction forces inherent from the contact mode operation of standard CSAFM. A piezoelectric oscillator was mounted in the standard CSAFM contact mode nose of the AFM which induces an oscillation amplitude vibration to the cantilever, and hence, it modulates the tip-sample contact force. This modulation frequency was selected as the resonant frequency of the cantilever (Rocky Mountain Nanotechnology RMN-25PT200-H), at 100 kHz frequency. This frequency is much higher than that of the cut-off frequency of the *I*-to-*V* converter (Resiscope, CSI Instruments), ensuring that the current is not modulated by the resonance frequency. In order to maximize the time that the tip remains in contact with the sample, we scanned the sample with parameters similar to the Small-Amplitude Small-Setpoint (SASS) case, which enables us to obtain high spatial resolution current images with minimal sample disturbance, good tip-sample electrical contact and fast acquisition times.

Raman spectra were acquired using a WITec alpha300 RA confocal Raman microscope using 785 nm solid-state laser excitation. Diffraction limited resolution images were taken in order to verify the CNT homogeneity. Overall, the samples exhibited homogeneous distribution of chiralities throughout. Depth profiles were measured by confocally scanning the depth using a piezoelectric stage.

Optical transmission was measured using both a Bruker HYPERION FTIR microscope connected to a VERTEX 70 spectrometer and an Ocean Optics ISP-REF integrating sphere and a FLAME-S-VIS-NIR-ES spectrometer, the latter of which has the smaller spectral range.

## Conflicts of interest

The authors declare no conflicts of interest.

## Supplementary Material

Supplementary information
