# Supplementary material for: Farming thermoelectric paper
Source: Energy Environ Sci. 2019 Jan 22;12(2):716–26. doi: 10.1039/c8ee03112f (PMC6394882; doi:10.1039/c8ee03112f)
Supplement: Supplementary file 1 [file EE-012-C8EE03112F-s001.pdf]

Electronic Supplementary Material (ESI) for Energy & Environmental Science.
This journal is © The Royal Society of Chemistry 2018

Supplementary information

Farming thermoelectric paper

Deyaa Abol-Fotouh[1,2,*], Bernhard Dörling [1,*], Osnat Zapata-Arteaga [1], Xabier Rodríguez-Martínez[1], Andrés Gómez[1], J. Sebastian Reparaz[1], Anna Laromaine[1,**], Anna Roig[1,**], Mariano Campoy-Quiles[1**]

[1] Institute of Materials Science of Barcelona (ICMAB-CSIC), Campus of the UAB, Bellaterra, 08193, Spain

[2] City of Scientific Research and Technological Applications (SRTA-City), New Borg Al-Arab, 21934, Egypt

Preparation of non-BC Reference Samples

CNT dispersions

SG65i CoMoCAT single-walled carbon nanotubes (CNTs) were purchased from Southwest NanoTechnologies and dispersed in 1,2-Dichlorobenzene (99%, Sigma-Aldrich) by bath sonication for 4 h at an initial concentration of 0.045 $mgml^{-1}$. The obtained dispersion was further centrifuged at 4000 rpm for 30 min and the supernatant was collected for later use.

Vacuum filtration

Volumes of 5 and 10 mL of the CNT supernatant were filtered through a vacuum filtration system using a 0.22 µm PVDF filter membrane. The film was dried at 80 °C and peeled off from the filter membrane before being dried again at 120 °C for 30 min.

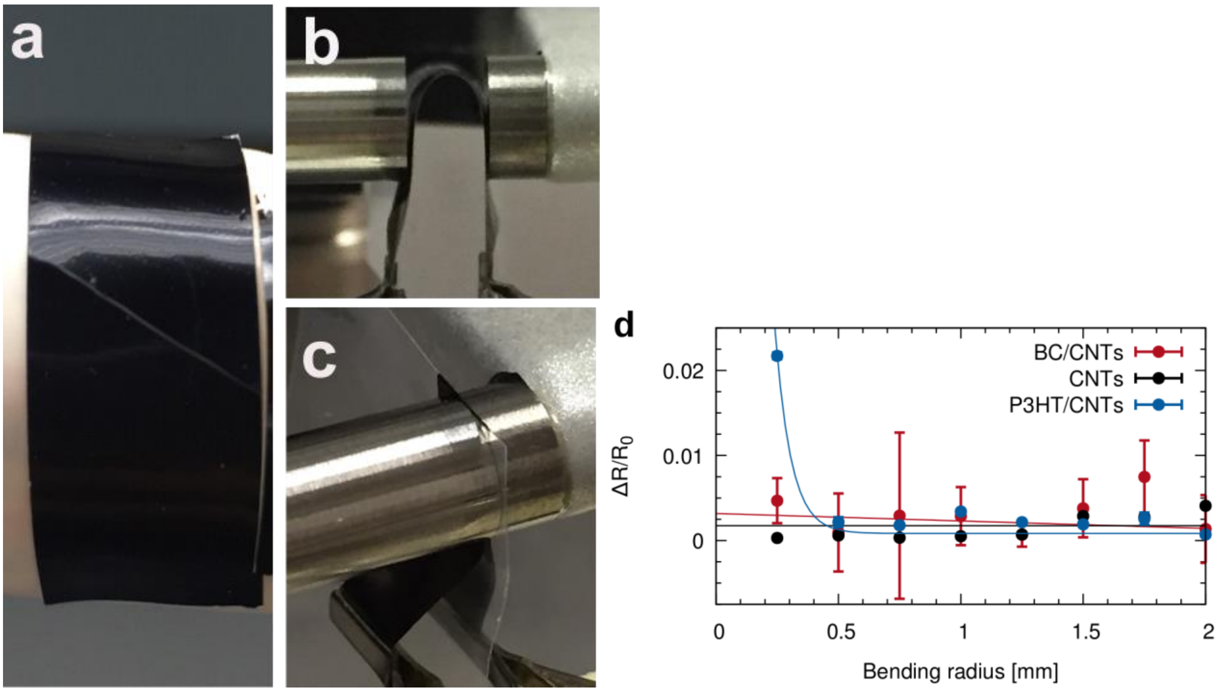

Supplementary Figure 1. Measurement of resistance change upon bending. (**a**) Ethanol treated PEDOT:PSS sample over a 7 mm bending radius, seen from the top. A clear crack is visible along the width of the film. (**b**) Samples were clamped in a micrometer to control the bending radius. A 50 µm PET film was placed beneath the sample, to serve as support for the free-standing films, while the electrical resistance was measured at the two electrodes at the ends of the film. (**c**) Composite sample at the lowest radius, a completely folded sample separated by a 50 µm PET film. (d) P3HT/CNT composites show a significant increase in resistance at the smallest bending radius, while neat CNTs and BC composites remain unaffected. The error bars correspond to the standard deviation over 100 measurements.

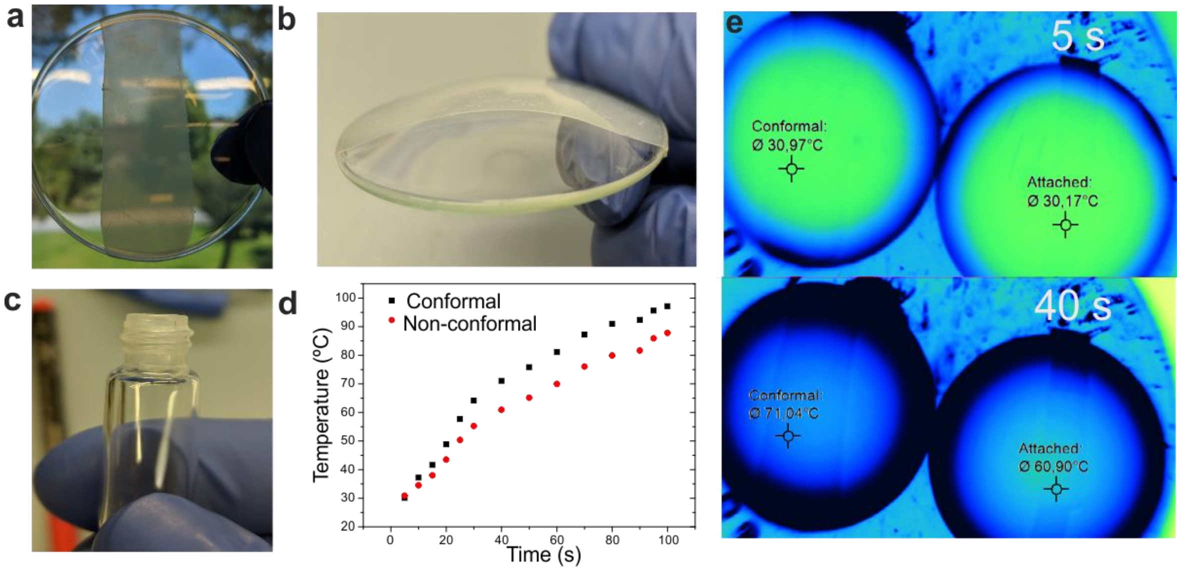

Supplementary Figure 2. Temperature rise of conformally fixed BC. (**a**) Top view of conformal BC fixed on glass. (**b**) Lateral view of conformal BC fixed on glass. (**c**) Harvested BC can be dried on intricate surfaces, resulting in a film wrapped permanently around the object. (**d**) Rise in temperature vs time. Conformal and non-conformal samples placed on a preheated hotplate, non-conformal sample was a free-standing film fixed at the edges with adhesive tape. After 100 s, a difference in temperature of 10 °C shows that conformal cellulose increases in temperature faster than non-conformal one. (**e**) Infrared pictures at time 5 and 40 s.

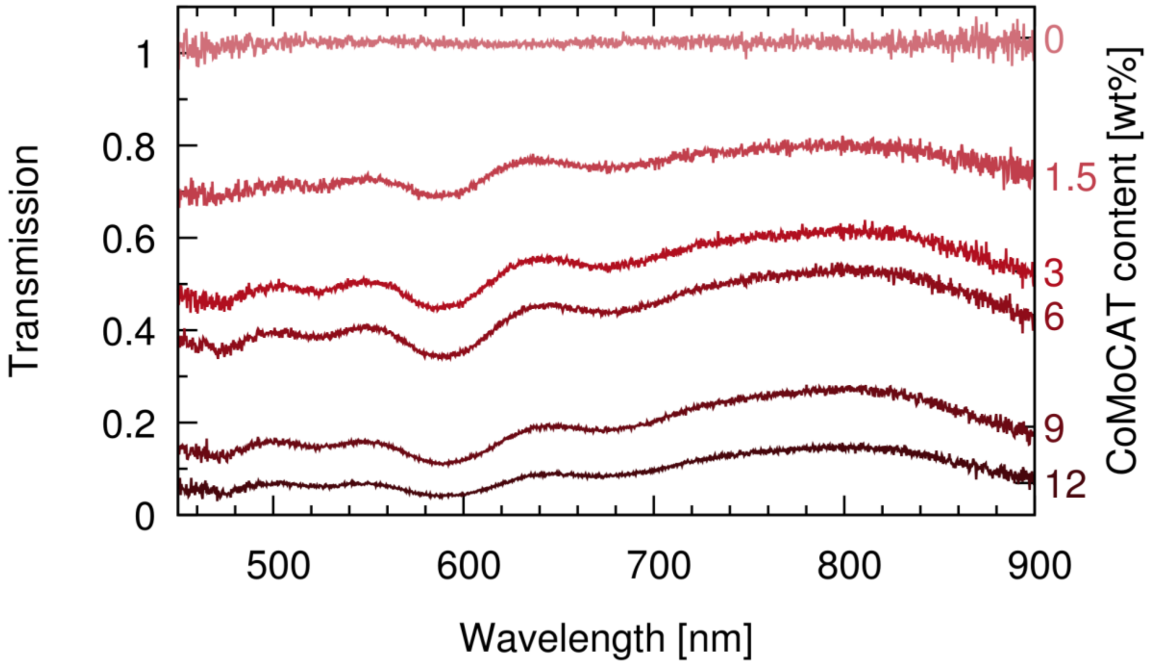

Supplementary Figure 3. Transmission measurements with an integrating sphere of BC and BC/CoMoCAT composites at different loadings.

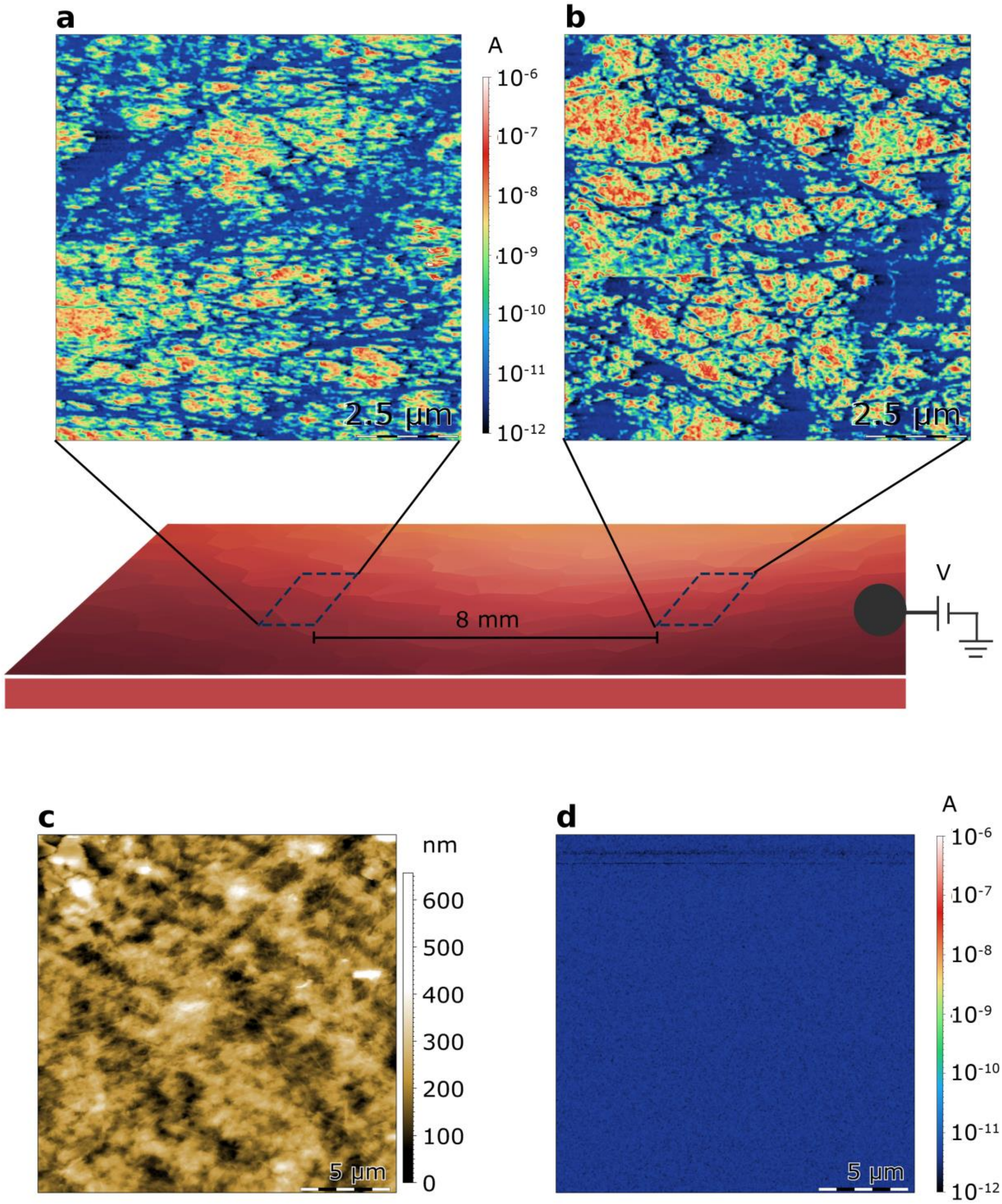

Supplementary Figure 4. Conductive AFM map for a BC/CoMoCAT40 sample measured at two different distances *d* from the ground electrode. (a) *d* = 10 mm. (**b**) *d* = 2 mm. (c) Topography and (d) current map of the non-conductive backside of the same sample.

One Laser Raman Thermometry

One laser Raman thermometry (1LRT) experiments were performed in backscattering geometry using a LabRam HR800 spectrometer with a grating of 1800 lines per millimeter. The setup is equipped with a liquid-nitrogen cooled charge coupled device (CCD) detector. A 488 nm line from an Ar^+-ion gas laser was used as excitation source. A 50x long working distance Olympus objective (NA 0.50) was used to focus the laser light yielding an intensity Gaussian beam of 2.2 µm in diameter; the beam size was determined by scanning the spot across a cleaved silicon edge with a high resolution motorized stage. The thickness of the samples was measured with a digital micrometer with an accuracy of ± 0.5 µm.

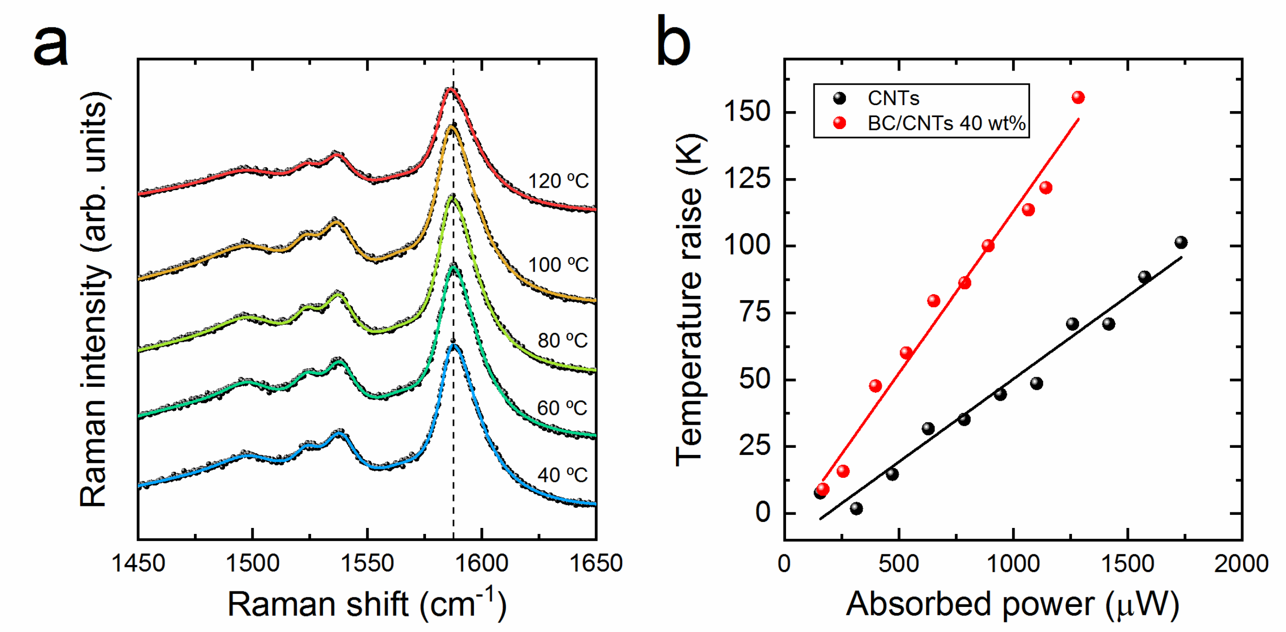

Supplementary Figure 5. (a) Calibration of the Raman shift with temperature for a CoMoCAT free-standing sample. (b) One laser Raman thermometry data for free-standing films of CoMoCAT (black circles) and one representative BC/CoMoCAT composite (red).

The free-standing films were held by a metallic ring and loaded onto a Linkam cryostat in ultra-high vacuum ($P < 10^{-5}$ hPa) to suppress convection and potential sample degradation. The thermal

contact of the samples with the Linkam hot stage was assured using silicon oil at the interfaces with the metal ring. Prior to starting the 1LRT experiments, the composite samples were baked at 120 ºC for 20-30 minutes to remove any water absorbed by the bacterial cellulose, which is known to be hygroscopic. The determination of the Raman shift coefficients was done right after the baking, while cooling back down from 120 to 30 ºC in steps of 10-20 ºC (Supplementary Figure 5a). The power absorbed was determined by measuring the transmitted and reflected components for each incident power selected. The transmitted power was measured by coupling a power meter to the backside of the Linkam stage in a home-made setup that assured perfect sealing. The reflected component was measured by collecting the reflected laser intensity at the CCD and then using a mirror of known reflectivity as calibration guideline. Both the transmitted and incident powers also required calibration and correction for the reflection at the Linkam windows.

The heat equation was solved in COMSOL Multiphysics using cylindrical 2D symmetry in models of at least 2 mm lateral size. Radiation was included in the calculations, which required the experimental determination of the emissivity. For this, we used an Optris PI450 infrared camera finding a very close value for all CNT containing samples (ε = 0.94). The temperature raise was also weighted by the Gaussian shape of the heat source (i.e. the laser) to take into account the collection yield of the Raman-scattered light. While keeping the rest of model inputs fixed, the thermal conductivity was swept in the range of absorbed laser powers used in each experiment (typically 20-400 μW) leading to a linear increase of the temperature raise as a function of the absorbed power (Supplementary Figure 5b). Since the slope inversely depends on the thermal conductivity, the thermal conductivity of the samples was determined by fitting the results of the simulation to the experimentally found trend.

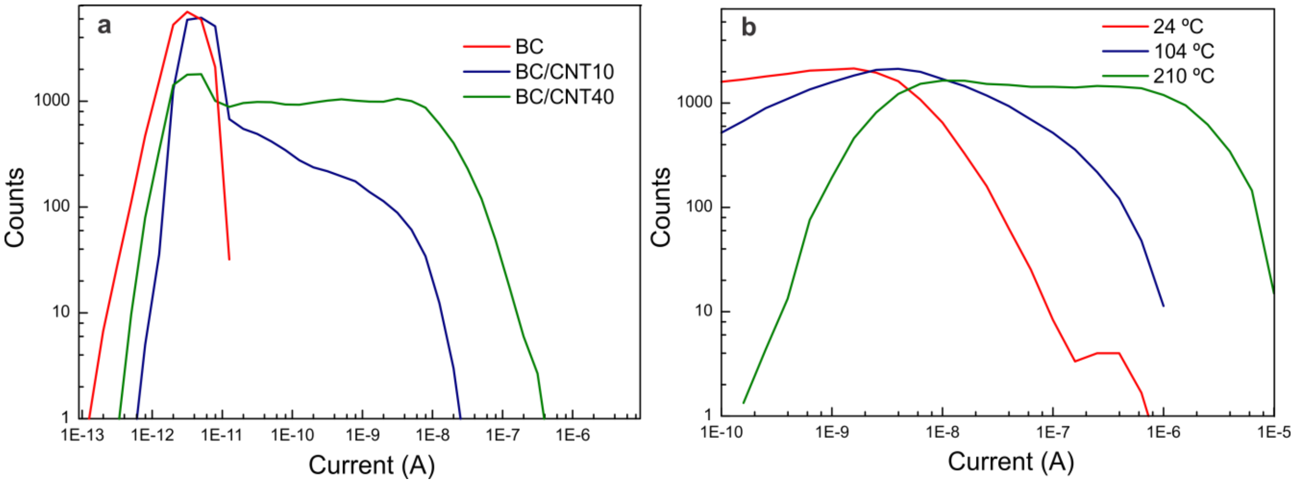

Supplementary Figure 6. Histograms of the conductive AFM maps for different CoMoCAT loading (**a**), and at different temperatures (**b**).

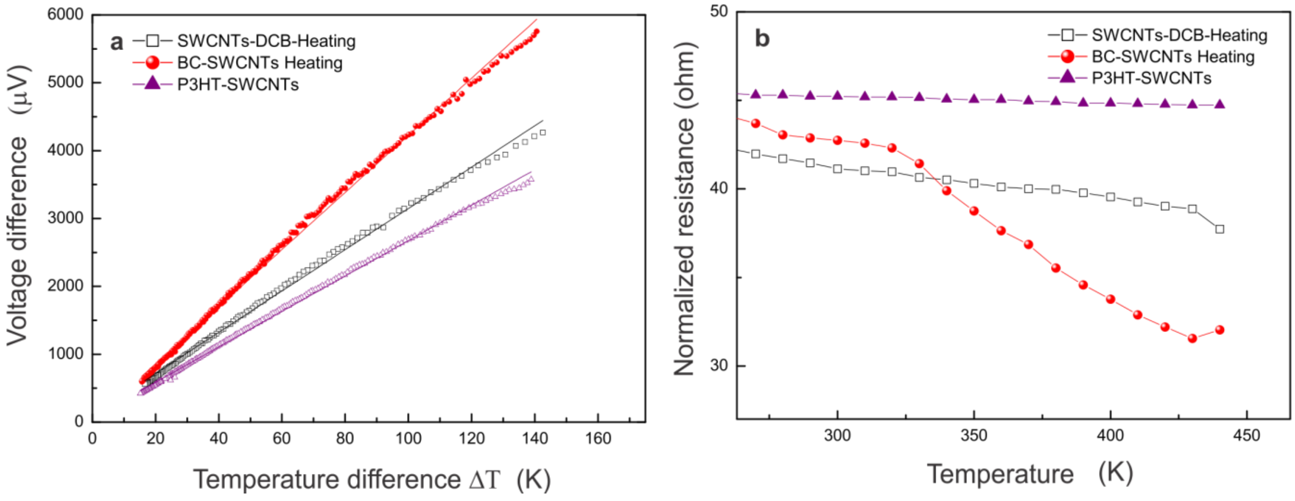

Supplementary Figure 7. Performance with temperature. (**a**) Seebeck voltage measured over an extended thermal gradient, shows linear behavior over a large range, and no changes upon repeated measurements, indicating good thermal stability for all samples. (**b**) Normalized resistance over temperature for all composites. A general semiconductive trend is observed for all samples.

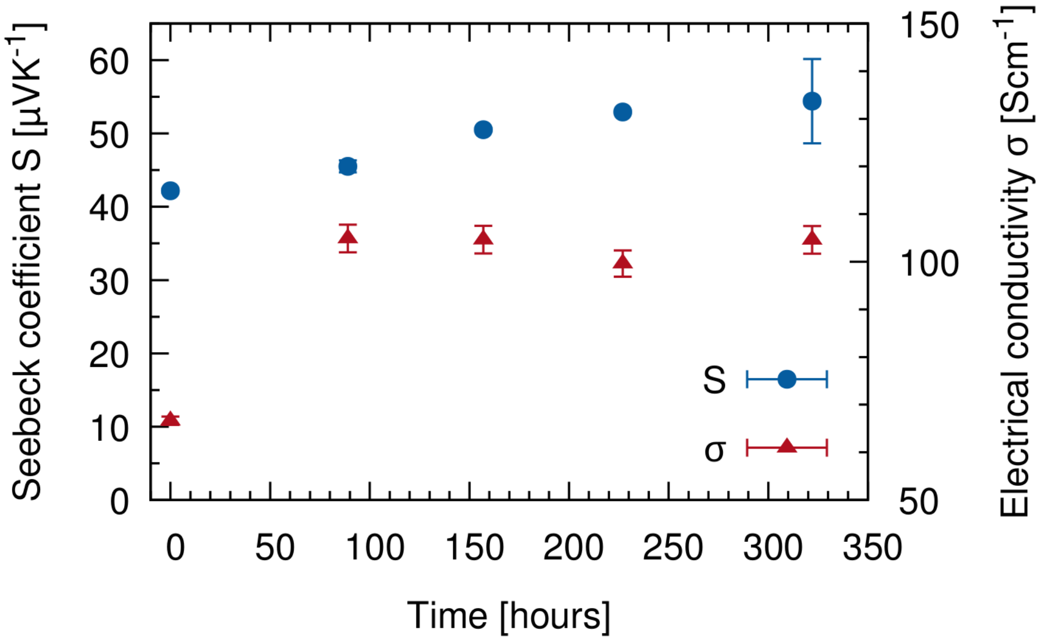

Supplementary Figure 8. Heat stability of BC/eDIPS samples at elevated temperatures. Samples were annealed at 200°C for nearly 350 hours with no significant change in electrical conductivity, apart from an initial increase due to the desorption of water. The Seebeck coefficient slightly increases with increasing annealing time.

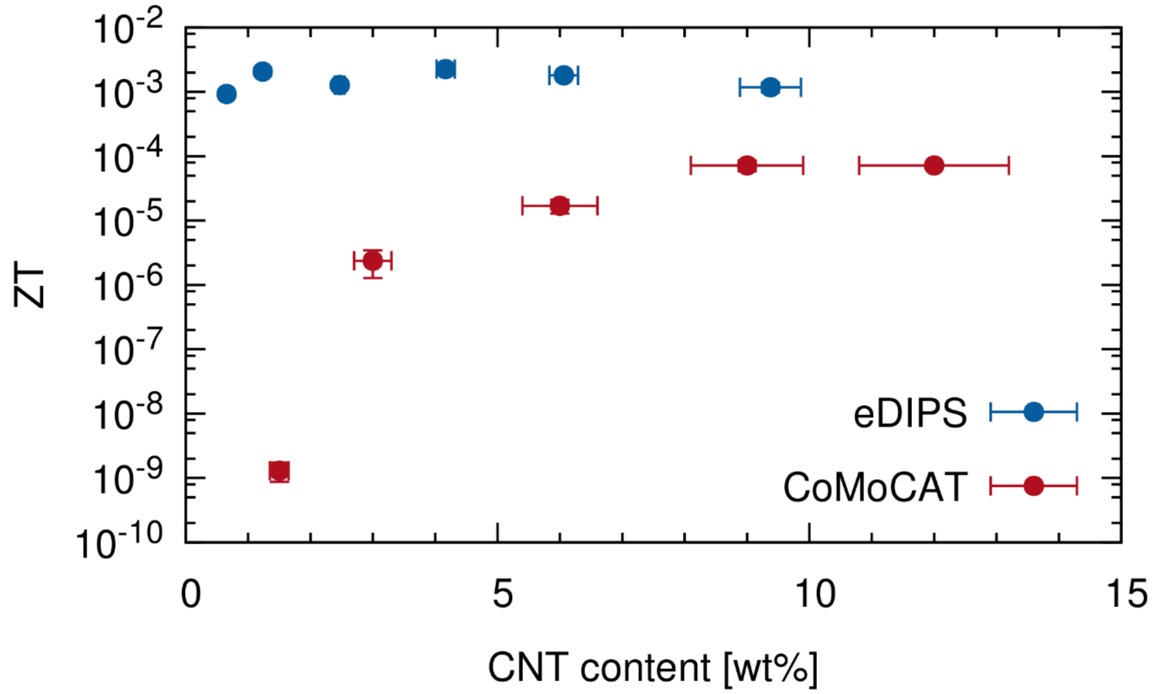

Supplementary Figure 9. ZT versus CNT content.

3ω method

The thermal conductivity of the bacterial cellulose (BC) was measured using the 3ω technique on thick films (>5 µm) transferred onto glass substrates. The 3ω technique is based on electrically heating a thin planar resistor using an AC harmonic current I_0 at a frequency ω, and subsequently measuring the resultant voltage drop at the first (V_ω) and third ($V_{3\omega}$) harmonics. By defining the normalized temperature coefficient of resistance as $\beta \equiv (1/R_0)[\partial R/\partial T]$ with R_0 the resistance of the resistor at the temperature T_0, the amplitude of the AC component of the temperature oscillations induced can be determined as

$$\Delta T_{AC} = \frac{2|V_{3\omega}|}{\beta|V_\omega|}$$

By solving the 2D heat equation for the geometry of a linear heat source supported on a semi-infinite medium, the thermal conductivity κ of such medium can be obtained as

$$\kappa = -\frac{P_0}{2\pi l}\frac{1}{(\partial\Delta T_{AC}/\partial \ln 2\omega)}$$

With P_0 the total dissipated power at the resistor and l the length of the resistor.

Since the AC current frequency determines the thermal penetration depth according to $1/q = \sqrt{\alpha/i2\omega}$, for thick films supported on a semi-infinite medium (i.e. a substrate) the ΔT_{AC} vs $\ln 2\omega$ curve shows low and mid-frequency regimes that primarily correspond to the substrate and the supported film, respectively. From the slope $(\partial\Delta T_{AC}/\partial \ln 2\omega)$ of both regions it is straightforward to determine their thermal conductivity.

Statistics performed on 7 different resistors thermally evaporated on glass-supported BC films yielded an average thermal conductivity of (0.52 ± 0.05) W m^{-1} K^{-1}, which is in very good agreement with the theoretical values reported elsewhere.

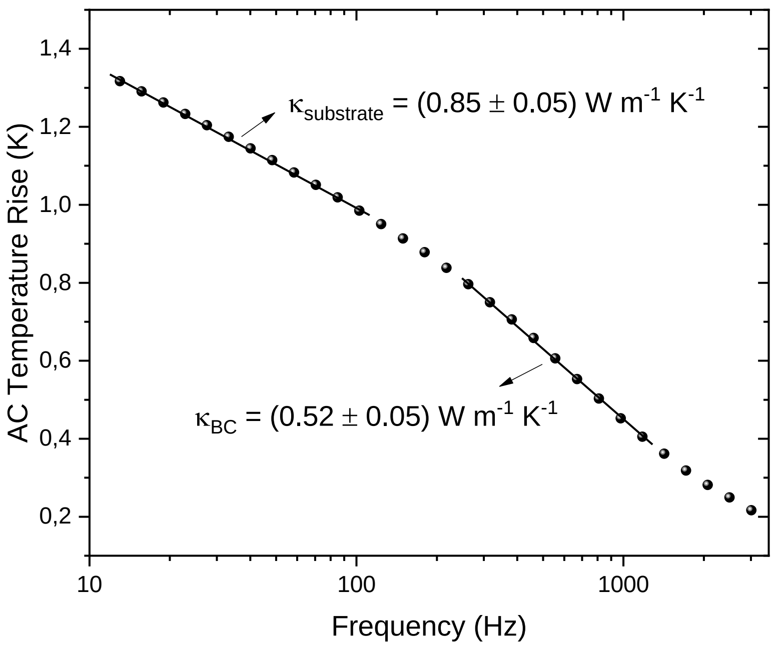

Supplementary Figure 10. Temperature rise versus heating frequency for a BC film on a glass substrate.
